# Titanium and Other Metal Hypersensitivity Diagnosed by MELISA® Test: Follow-Up Study

**DOI:** 10.1155/2021/5512091

**Published:** 2021-06-03

**Authors:** Radka Vrbova, Stepan Podzimek, Lucie Himmlova, Adela Roubickova, Marketa Janovska, Tatjana Janatova, Martin Bartos, Alex Vinsu

**Affiliations:** Institute of Dental Medicine, First Faculty of Medicine and General University Hospital in Prague, Charles University, Prague 121 11, Czech Republic

## Abstract

This study is aimed at proving the clinical benefit of the MELISA® test in the minimization or complete elimination of health problems in patients with confirmed hypersensitivity to metals used for tissue replacements. A group of 305 patients aged 20-75 years with previously proven metal hypersensitivity (initial MELISA® test), mainly to titanium and then to another fifteen metals, was chosen from the database at the Institute of Dental Medicine. From these patients, a final group of 42 patients agreed to participate in the study, 35 of which were female and 7 were male. The patients completed a special questionnaire aimed at information regarding change of health status from their last visit and determining whether the results of the initial MELISA® test and recommendations based on it were beneficial for patients or not. They were clinically examined, and peripheral blood samples were taken to perform follow-up MELISA® tests. Questionnaire data was processed, and the follow-up MELISA® test results were compared with the results of the initial MELISA® tests. For statistical analysis, the Fisher's exact test and paired *T*-test were used. Thirty-two patients reported that they followed the recommendations based on the results of the initial MELISA® tests, and of these, 30 patients (94%) confirmed significant health improvement. Six patients did not follow the recommendation, and from these, only one patient reported an improvement in his health problems. By comparison of the initial and follow-up MELISA® test results, it can be stated that the hypersensitivity to the given metal decreased or disappeared after the therapeutic interventions performed based on the initial MELISA® test results. The evaluation of the data obtained from patients in this study confirmed a significant clinical benefit of MELISA® test.

## 1. Introduction

Metals, such as cobalt-chromium-molybdenum alloys, stainless steel, titanium and its alloys, and tantalum, are well known and are widely and successfully applied in current implantology and prosthetics. Although the materials differ more or less in their physicochemical and mechanical properties, they are all considered biocompatible and suitable for use in the human body. It is well known that the surface of an implant is very important for body reaction. A lot of studies have been published on surface treatments with many being incorporated by the implant manufacturers into clinical practice (orthopaedics, prosthetics, dental implantology, etc.), between typical applied surface treatments belong to the mechanical process of sandblasting, the chemical process of acid etching (or a combination of both), plasma-sprayed coatings, or the use of 3D printing technology for trabecular structure creation. Other physical or chemical surface treatments are examined and reviewed by a number of research teams. The interesting and promising chemical treatment is surface functionalization by biomolecules such as proteins, peptides, or peptidomimetics as coatings, where a higher probability of successful bone-implant integration is predicted [1] or functionalization by calcium phosphate thin layers, which leads to increasing cell activity and collagen formation in comparison to inert substrates [2–5]. The traditional plasma-sprayed application of thin CaP layers has its main limitations in nonhomogeneous porosity, weak adherence to metal substrates, degradation and delamination during long-term function [6, 7], or in low cohesive strength depending on the layer thickness [8]. The abovementioned facts have led to research on the deposition of bioactive materials using advanced physical and chemical techniques such as the hydrothermal process [9, 10], the hybrid technique of magnetron sputtering and thermal methods [11], and sol-gel and biomimetic precipitation or ion beam assisted deposition (IBAD) [12–15]. The advantages of the latter technique are the possibility of functionally graded coatings, which can enhance osseointegration in the early stages following implantation [12, 13], bonding to the substrate on an atomic level as a result of intermixing of the coating and substrate atoms during the ion beam process, the possibility of antimicrobial admixtures deposition, for example, silver [16] or copper ions [17], or the deposition of nitride and carbon ions causing the creation of more mechanically resistant implant surfaces in comparison to surfaces without this treatment [18–20].

Despite all of the advantages and optimizations of the surface treatments and techniques mentioned above, the implant is a foreign material for the human organism and its long-term influence on the recipient's body, particularly to the immune system, remains to be fully understood. In general, the implanted materials are well tolerated by the patients. Those who are more at risk include individuals with diagnosed allergies, history of metal hypersensitivity, asthma, or autoimmune diseases. The hypersensitive reactions of the human organism are supported by corrosion or by the wear processes of replacements, with subsequent release of metallic ions into the tissue. The released ions can form complexes with endogenous proteins and activate the immune system, induce a local inflammatory reaction, or damage the bone structure [21–25]. Chronic inflammation following the occurrence of wear particles has been recognized as the main biological mechanism leading to implant failure [26–28]. The most common metal sensitizers are nickel, cobalt, and chromium [25, 29–31] that are components of cobalt-chromium-molybdenum alloys and stainless steel. Of all the metals used in implantology, titanium is currently regarded as a material of choice for its biocompatibility, osseointegration capability, and corrosion resistance ensured by the presence of a stable passivation surface layer [32–34]. The cytotoxicity of titanium has not been confirmed [35, 36], but on the other hand, publications with the opposite outcome have also been found [37, 38]. The insertion of a titanium implant leads to increased exposure of the organism to this metal. Titanium ions can be found in the tissue surrounding the implant or in lymphatic nodes. The titanium debris is also present in the lysosomes of macrophages, which may lead to the hypersensitivity reactions of type IV [23, 33, 39–41]. Cases detecting high amounts of titanium [23] and pigmentation in the vicinity of implants or in organs [33] were described. Hypersensitivity reactions are associated with very unpleasant impacts on the health of patients. There are two main types of tissue reactions to released ions: nonspecific granulomatous reactions mediated by macrophages and the lymphocyte responses with a predominance of T-lymphocytes releasing cytokines. Tissue reactions are described according to predominant cellular response as either macrophage-dominated type without immunological memory or lymphocyte-dominated type, describing a T-lymphocyte-mediated reaction characterized by an immunological memory [42]. A variety of inflammatory mediators may be involved, such as cytokines (IL-1, IL-2, IL-4, IL-5, IL-6, IL-10, IL-13, IL-17, IFN-*γ*, and IP-10), chemokines (MIP-1*α* and MIP-1*β*), and growth factors (GM-CSF and PDGF) [42]. The diagnostic methods have been used to determine hypersensitivity reactions, including patch testing, lymphocyte transformation test (LTT), leukocyte migration inhibition test (LIF), lymphocyte activation test (LAT), and memory lymphocyte immune-stimulation assay (MELISA®) [29, 42–48]. Patch testing is the most commonly used diagnostic method for assessing hypersensitivity to various materials. However, it has recently been identified as a somewhat controversial method [49–51]. Similarly, the study of Granchi et al. found significantly increased frequency of positive patch test reactions after total knee arthroplasties; however, expected number of loosened prostheses in studied patients was not confirmed [26]. LTT tests the immune components responsible for the hypersensitivity more directly than patch testing, as the antigen-presenting cells in the skin are not present in the deeper tissues [52]. Memory lymphocyte immune-stimulation assay (MELISA®) is based on the evaluation of the proliferation of peripheral blood lymphocytes in vitro after incubation with metal ions. This test modification for assessment of hypersensitivity reactions to various metals or nonmetal materials is used in many laboratories [30, 43, 53–58]. MELISA® test could help the surgeon in selecting the most appropriate and tolerated implant material for the patients and may benefit the performance of the implantation by decreasing postoperative complications or revision surgeries.

Symptoms of hypersensitivity reaction include pain, joint effusion, swelling or allergic dermatitis (localized or systemic), changes in the oral mucosa and mucosal immune system connected with the presence of dental implants or prosthetic work [59], delayed bone healing, implant instability, and ultimately the aseptic failure of implants [60–62]. Due to previously described serious symptoms and the increasing incidence of hypersensitivity reactions to the implant materials in the population in recent years [63–70], this study is aimed at demonstrating the benefits of the MELISA® test for the minimization or complete elimination of health problems in patients with confirmed hypersensitivity to metals. The hypothesis of this study is that compliance with the health recommendations based on the results of MELISA® test will improve patients' symptoms induced by metal hypersensitivity.

## 2. Materials and Methods

### 2.1. Description and Selection of Patients

A group of 305 patients with previously proven metal hypersensitivity was chosen from the database at the Institute of Dental Medicine. Inclusion criteria were as follows: age between 20 and 75 years, proven hypersensitivity to titanium, area of the capital city and its immediate surroundings, and patients with health symptoms connected with their oral cavity metal restorations, endoprosthesis, or with osteosynthesis devices. Symptoms found in oral cavity were changes of oral mucosa such as pigmentations, presence of aphthae and lichen, painful blisters and gingival hyperplasia, and other symptoms such as swelling and burning accompanied by dry oral cavity and feeling of metallic taste. In patients with orthopaedic endoprostheses, swelling, pain, aseptic loosening, and skin defects were presented. All these symptoms were often associated with chronic fatigue, and the overall health of patients was very uncomfortable and life-limiting. Exclusion criteria were as follows: autoimmune disease and/or immunosuppressive therapy. Based on the inclusion and exclusion criteria, the subset of 93 patients was created. These patients were asked to participate in the study via mail, and a final group of 42 patients, 35 females and 7 males aged between 25 and 75 years with previously diagnosed hypersensitivity to metals by MELISA® test, agreed to their participation. The study was performed in agreement with the Helsinki Declaration after approval by the Ethical Commission of the General University Hospital in Prague.

### 2.2. Clinical Examination

After signing the informed consent, the patients were clinically examined by three clinicians using precoordinated procedure for the detection and recording of metals present in their oral cavity and the patients were asked to fill a special questionnaire. This questionnaire focused on exposure to metals with questions regarding family history, exposure to metals in the past/present (employment, tattoos, and objects in direct/indirect contact with skin—jewellery, piercings, watches, and presence of implants in the body of patients), allergy occurrence, and smoking experience. Ten items closely related to the relevant concept of the study were selected for the purpose of the questionnaire evaluation (Table 1). The questionnaires were evaluated focusing on the patient's health status since their last visit and especially on the benefit/nonbenefit of the in vitro MELISA® test. The main purpose of the questionnaire was to find out whether the recommendations based on the results of the initial MELISA® test had a significant effect or not. For this purpose, the key questions were focused on the utilization of MELISA® test results and the recommendations based on it to eliminate/minimize the patient's health complications. The patients were especially advised to the following: nonmetallic remediation or elimination of exposure to risk metals and use of nonmetallic or risk-free metal implants in the first or revision surgery.

### 2.3. MELISA® Test

MELISA® test, validated by an independent laboratory [43], was used in this study for assessment of metal hypersensitivity reactions. The follow-up MELISA® test was performed in 42 patients after signing the informed consent. Peripheral blood (45 ml) was taken from the patients. T-lymphocytes were separated by Ficoll-Paque gradient centrifugation. Autologous serum was used for the cultivation of lymphocytes. T-lymphocytes were cultivated with metal ions for 5 days in an atmosphere of 5% carbon dioxide in humidified air at 37°C. Control T-lymphocyte cultures were incubated under the same conditions in medium only. As a positive control, T-lymphocytes were cultivated with pokeweed mitogen. After 5 days, T-lymphocyte proliferation was measured using the radioactive ^3^H thymidine incorporation. The rate of T-lymphocyte proliferation in stimulated cultures was compared to the rate in nonstimulated cultures and evaluated by stimulation index (SI): counts per minute (cpm) in metal-treated cultures divided by the mean cpm of the control cultures [56, 71]. A stimulation index less (SI) than 2 was regarded as a negative reaction, SI 2.01-4 as a weakly positive reaction, SI 4.01-10 as a positive reaction, and SI higher than 10 as a strongly positive reaction.

### 2.4. Statistical Analysis

Data from the questionnaires was statistically analysed using Fisher's exact test, and the results of the initial and follow-up MELISA® tests were compared and statistically analysed using paired *T*-test.

## 3. Results

From the group of 42 patients, 38 questionnaires were evaluable with the remaining 4 questionnaires not answered responsibly by patients. Two of these patients underwent the follow-up MELISA® test shortly after the initial test (after half a year), so they did not have enough time to solve their situation. One of the patients did not answer the question regarding the benefit of the MELISA® test, and the last one of the 4 patients was problem-free; only the determination of hypersensitivity to metals was required before the planned implant procedure from the preventive reasons. In the group of 38 patients, there were 4 patients with polyvalent allergy, 5 patients with health problems after orthopaedic surgery (inflammation, swelling, pain, itching, dermatitis, and loss of joint function), 1 patient with health problems after endovascular coiling (chronic fatigue and tinnitus), 25 patients with symptoms in the oral cavity (oral lichen planus, burning, itching, gingival hyperplasia, blisters, aphthous stomatitis, and dental implant rejection), and 3 patients with other health problems (chronic fatigue, headache and spine pain, atopic eczema, limb pain, and swelling). Significant health improvement was confirmed in 30 patients out of 32 patients who followed recommendations based on the results of the initial MELISA® (Table 2). The significant differences were statistically analysed using Fisher's exact test (*P* = 0.0002).

**Figure 1:**
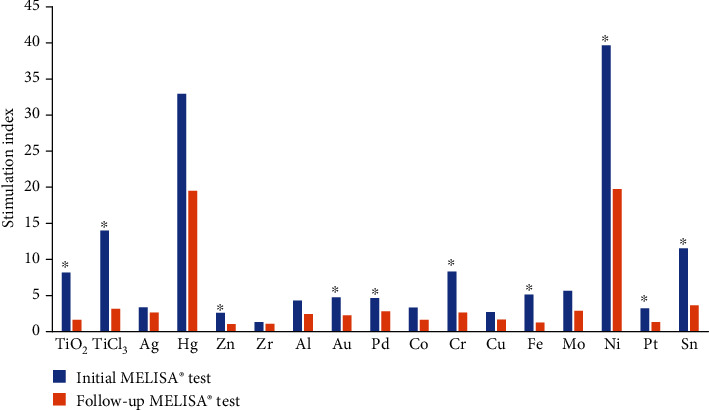
The results of the initial and follow-up MELISA® tests; ^∗^significant decrease (*P* < 0.05, paired *T*-test).

The mean values of the stimulation index for each tested metal in the group of 42 patients are shown in Figure 1. The blue columns represent the results of initial MELISA® tests, and the orange columns belong to the results of follow-up MELISA® tests. From this figure, it is obvious that the elimination of exposure to risk materials, be it dental, orthopaedic, or other, which exhibit hypersensitivity of the patient to them, resulted in a decrease of all values of the SI, with a significant decrease found in 10 metals. The highest values of SI were obtained for nickel (Ni), mercury (Hg), titanium trichloride (TiCl_3_) and tin (Sn) (strongly positive reaction), for chromium (Cr), titanium dioxide (TiO_2_), molybdenum (Mo), iron (Fe), gold (Au), palladium (Pd) and aluminium (Al) (positive reaction), lower values of SI for silver (Ag), cobalt (Co), platinum (Pt), copper (Cu) and zinc (Zn) (weakly positive reaction), and the lowest values of SI for zirconium (Zr) (negative reaction). The values of SI for Ni and Hg are distinctly higher than for other metals, and the hypersensitivity to these metals was found in most patients (Figure 1).

**Figure 2:**
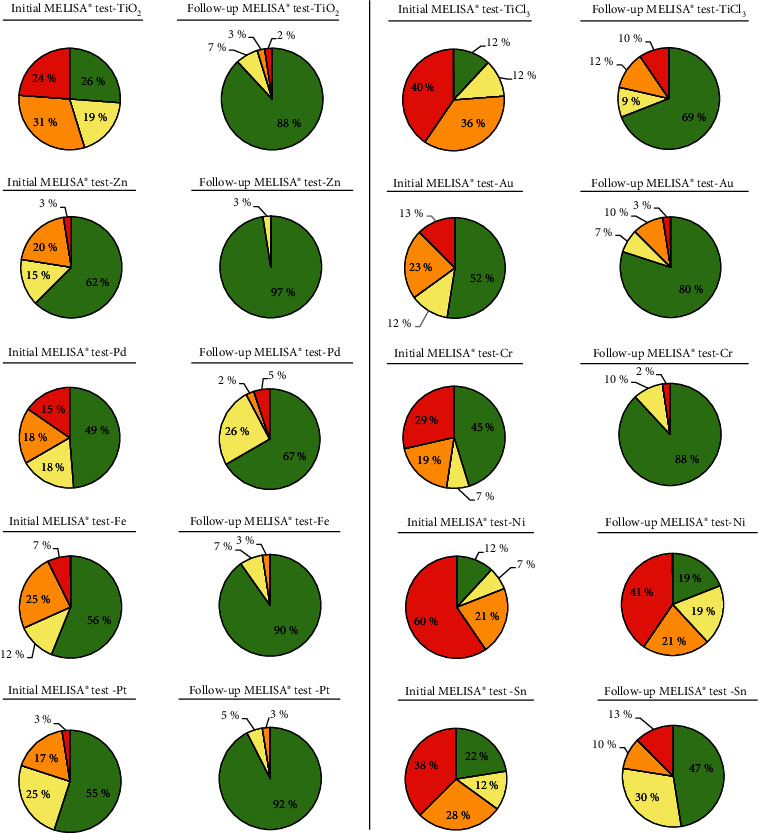
Percentage of patients in individual intervals of the stimulation index: green ≤ 2.0, yellow = 2.1‐4, orange = 4.1‐10.0, and red ≥ 10.1; comparison of the initial with the follow-up values of the stimulation index—metals with significant differences.

**Figure 3:**
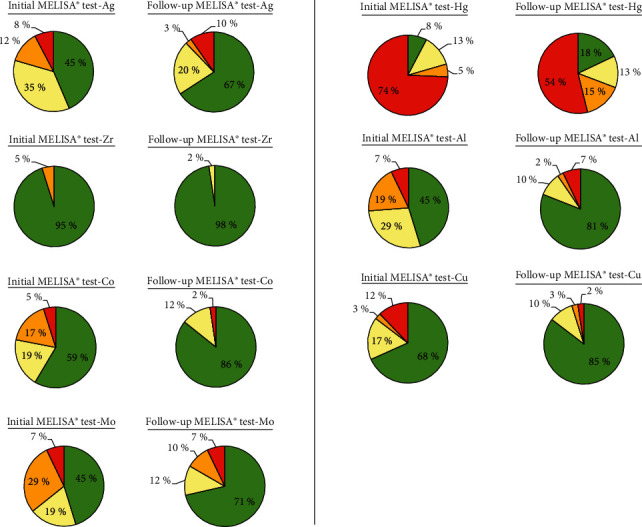
Percentage of patients in individual intervals of the stimulation index: green ≤ 2.0, yellow = 2.1–4, orange = 4.1–10.0, and red ≥ 10.1; comparison of the initial with the follow-up values of the stimulation index—metals with nonsignificant differences.

Figures 2 and 3 show for each tested metal the percentage of patients with strongly positive, positive, weakly positive, and negative reaction in the initial MELISA® test (see Figures 2(a) and 3(a)) and in the follow-up MELISA® test (see Figures 2(b) and 3(b)). The metals with significant differences (*P* < 0.05, paired *T*-test) are shown in Figure 2, and metals with nonsignificant differences are shown in Figure 3. In the follow-up MELISA® tests (Figures 2(b) and 3(b)), the enlargement of the green/yellow fields showing negative or only weakly positive reaction is clearly evident in all tested metals. The reduction or complete disappearance of the orange/red fields with the positive or strongly positive reaction is also clearly evident (Figure 2—Zn, Fe, Pt, and Cr; Figure 3—Zr and Co). Overall, it can be stated that for each tested metal, the hypersensitivity to the given metal decreased or was eliminated after therapeutic interventions were performed based on the results and issued recommendations from the initial MELISA® test.

## 4. Discussion

Delayed-type hypersensitivity immune response consists in the contact of T-cells with activated antigen, which leads to secretion of various cytokines activated by macrophages, monocytes, neutrophils, and other inflammatory cells [29, 72]. Advantages of the MELISA® test used in this study (compared to patch tests) are systemic hypersensitivity testing (compared to skin hypersensitivity testing by patch testing), wider panel of metals for testing (including titanium—accuracy of patch testing for titanium is variable), in vitro testing (compared to direct contact with possible allergens by patients during patch testing), standardized testing (compared to lack of standardization in patch testing), and no contraindications (compared to contraindications in patch testing—children, pregnant and lactating women, and patients with autoimmune and skin diseases).

In this study, thirty-two patients reportedly followed the recommendations based on the results of the initial MELISA® test issued to eliminate their health problems, and of these, 30 patients (94%) confirmed significant health improvement. Only six patients did not follow the recommendations, and from these, only 1 patient reported an improvement in his health problems. The benefits of the MELISA® test for the minimization of health problems in patients with hypersensitivity to metals were confirmed in this study. Whether or not the MELISA® test is clinically relevant for the detection and monitoring of metal sensitivity has also been investigated by team of Elizabeth Valentine-Thon. Their results confirmed remarkable clinical improvements in patients and significant reductions or complete normalization of specific lymphocyte reactivity after removal of the allergenic metals [43, 44].

In this study, from all tested metals, the highest level of hypersensitivity was found in reaction to nickel and mercury and the hypersensitivity to these two metals was found in most patients. This finding corresponds with common knowledge that metals such as nickel and mercury are very well-known allergens [73–76]. Significant decrease of stimulation index in MELISA® test was observed for nickel, but for mercury, only nonsignificant decrease was determined. Mercury is present in dental amalgams. On the basis of MELISA® test results, frequent recommendation for patients with health problems was the removal of amalgams and its replacement by dental composite resins. The nonsignificant decrease in mercury could be due to the fact that the patients changed the amalgams gradually, and in the time of follow-up examination, amalgam fillings were only partially replaced in a number of patients. Therefore, their hypersensitivity did not reach significantly low level before follow-up MELISA® testing was performed.

High level of hypersensitivity was found also in reaction to titanium. Nowadays, the hypersensitivity to titanium materials is recorded more frequently. This is probably due to the fact that titanium and its alloys currently belong to materials of choice for biological applications. People are exposed to titanium (primarily titanium dioxide) not only from medicine devices, but also from different sources such as watches, jewellery, body creams, make-ups, deodorants, toothpastes, and food. The current scientific literature summarizes the state of knowledge of titanium allergy in review articles and draws attention to the importance of this issue [70, 77]. The values of stimulation index obtained in our study have confirmed increased hypersensitivity to titanium (Figure 1—TiO_2_ and TiCl_3_). These values significantly decreased in follow-up MELISA® test. This is in concordance with recommendations to avoid exposure to this metal from all possible sources that was followed by studied patients. In addition, some orthopaedic patients underwent replacement/application of more suitable implant material, selected on the basis of MELISA® test results.

Compared to the metals with the highest hypersensitivity response, the lowest hypersensitivity was found for zirconium, which is a part of zirconium ceramics. It is well known that ceramic-based implants do not suffer from corrosion or degradation in biological environments as metals do, and therefore, ceramic materials may be a suitable alternative to metallic materials causing adverse reactions [78]. The results of our study support this claim by the lowest values of stimulation index and negative hypersensitivity reaction to zirconium in the majority of patients in both, the initial MELISA® test (95% of patients) and the follow-up MELISA® test (98% of patients). Zirconium, as the only studied metal, did not cause a strongly positive reaction in any of tested patients.

All tested metals are commonly used in dentistry (dental alloys, amalgam, and dental implants) and are also widely used in orthopaedic implants or osteosynthesis devices. The experience of our team from recent years has shown growing interest of orthopaedists in MELISA® testing due to the increased number of health problems after surgeries and a higher number of aseptic implant failures. Implants releasing metal ions or particles due to their wear and corrosion are more likely to induce metal hypersensitivity [25, 28, 72, 79]. Metal hypersensitivity to components of dental materials (most commonly to mercury, nickel, cobalt, chromium, gold, and palladium) led to oral diseases, such as oral lichen planus/lichenoid reactions, orofacial granulomatosis, cheilitis, perioral dermatitis, and burning mouth syndrome [80–82]. Metal hypersensitivity has been also associated with development of diverse diseases, such as nonischemic dilated cardiomyopathy [83], Takotsubo syndrome [57], connective tissue disease [56], fibromyalgia [84], and autoimmune thyroiditis [85]. In patients with diagnosed metal hypersensitivity and with health problems associated with metal medical devices, their health problems disappeared after the metal materials were removed [30, 65]. The important finding resulting from this study is that hypersensitivity to each tested metal decreased or was eliminated after therapeutic interventions that were performed based on the results and issued recommendations from the initial MELISA® test.

## 5. Conclusions

Metallic materials applied into the human organism may cause hypersensitivity. Metal hypersensitivity can be “in vitro” tested by MELISA® test. Based on the test results, patients are recommended a treatment procedure in order to reduce their health complications. The replacement or elimination of exposure to risk metals can lead to significant clinical improvement. The evaluation of the data obtained from patients in this study confirmed the hypothesis regarding the significant benefits of MELISA® test. Nevertheless, further follow-up studies with increased sample size should be performed for additional confirmation of our results. At present, there is no treatment guideline regarding a unified approach to the issue of hypersensitivity to metallic, but increasingly also to nonmetallic materials used in the human organism. This validated test for determination of immune hypersensitivity response to metal ions would be very suitable for reducing health complications in clinical practice due to the increase of hypersensitivity occurrence in population.

## Figures and Tables

**Table 1:** The main items of the questionnaire.

Items of questionnaire	Evaluation
Professional contact-exposure to metals	Yes/no, which metals/contact duration
Smoking experience	Yes/no, how much and how long
Used medicines	Yes/no, which
Implants	Yes/no, which and how long
Tattoos	Yes/no, how long, how big
Contact with jewellery, watches, piercings	Yes/no, burning, itching, swelling, rash, type of material
Blackening of metals	Yes/no, type of material
Allergy	Yes/no, specification
Utilization of initial MELISA® test results	Were the results of MELISA® test helpful? Yes/no
Treatment measures and recommendations based on the results of the initial MELISA® test	Yes/no, removal of metals from oral cavity, revision/exchange of implants

**Table 2:** The compliance with expert recommendations and improvement of health (number of patients).

		Improvement		
		Yes	No	Total
Recommendation was followed	Yes	30	2	32
	No	1	5	6
	Total	31	7	38

## Data Availability

The basic data can be requested from the author (radka.vrbova@vfn.cz).
